# Detection of Heteroplasmic Mitochondrial DNA in Single Mitochondria

**DOI:** 10.1371/journal.pone.0014359

**Published:** 2010-12-16

**Authors:** Joseph E. Reiner, Rani B. Kishore, Barbara C. Levin, Thomas Albanetti, Nicholas Boire, Ashley Knipe, Kristian Helmerson, Koren Holland Deckman

**Affiliations:** 1 Physical Measurement Laboratory, National Institute of Standards and Technology, Gaithersburg, Maryland, United States of America; 2 Material Measurement Laboratory, National Institute of Standards and Technology, Gaithersburg, Maryland, United States of America; 3 Department of Chemistry, Gettysburg College, Gettysburg, Pennsylvania, United States of America; RIKEN Brain Science Institution, Japan

## Abstract

**Background:**

Mitochondrial DNA (mtDNA) genome mutations can lead to energy and respiratory-related disorders like myoclonic epilepsy with ragged red fiber disease (MERRF), mitochondrial myopathy, encephalopathy, lactic acidosis and stroke (MELAS) syndrome, and Leber's hereditary optic neuropathy (LHON). It is not well understood what effect the distribution of mutated mtDNA throughout the mitochondrial matrix has on the development of mitochondrial-based disorders. Insight into this complex sub-cellular heterogeneity may further our understanding of the development of mitochondria-related diseases.

**Methodology:**

This work describes a method for isolating individual mitochondria from single cells and performing molecular analysis on that single mitochondrion's DNA. An optical tweezer extracts a single mitochondrion from a lysed human HL-60 cell. Then a micron-sized femtopipette tip captures the mitochondrion for subsequent analysis. Multiple rounds of conventional DNA amplification and standard sequencing methods enable the detection of a heteroplasmic mixture in the mtDNA from a single mitochondrion.

**Significance:**

Molecular analysis of mtDNA from the individually extracted mitochondrion demonstrates that a heteroplasmy is present in single mitochondria at various ratios consistent with the 50/50 heteroplasmy ratio found in single cells that contain multiple mitochondria.

## Introduction

Viral pathogenesis, tumor genesis and metastasis, progression of genetic disorders, and apoptosis are complex biological processes that rely on multiple biochemical signals to define the cell physiology. Gene expression levels and covalent modification of biomolecules are just two types of biochemical responses that lead to cellular heterogeneity, which may trigger variations in pathogenic steps like viral infection or variable drug responsiveness. As recently discussed by Snijder *et al.* [1], the pathway by which a disease develops is not always clear, therefore novel methods are needed to characterize subtle differences between cells. These methods could help explain disease development, which in turn could lead to new detection methods and therapies.

A growing number of diseases have been linked to mutations within the mitochondrial genome. Examples of these mitochondria-based diseases include myoclonic epilepsy with ragged red fiber disease (MERRF), mitochondrial myopathy, encephalopathy, lactic acidosis and stroke (MELAS) syndrome, and Leber's hereditary optic neuropathy (LHON) [2], [3], [4]. It is generally believed that the severity of a mitochondrial disorder is related to the percentage of mutant mtDNA within a given cell and tissue [5], [6].

A dynamic picture of mitochondria in mammalian cells has emerged where the mitochondrial genome is impacted via direct mitochondrial mixing [7]. Little is known about the resulting distribution of mutated mtDNA throughout the mitochondrial matrix and its corresponding effect on the development of mitochondria-related diseases. Studying mtDNA from individual mitochondria could help elucidate the connection between the intracellular distribution of mtDNA and the onset of a corresponding disease.

Analyzing mtDNA from single mitochondria requires methods for both isolation and analysis of individual mitochondria [8]. Cavelier *et al.* employed fluorescence microscopy and flow cytometry to isolate individual mitochondria, from a large aggregate of cells [9]. They observed both homoplasmy and heteroplasmy (the latter being a mixture of wild-type and mutated mtDNA containing a single MERRF point mutation) within single mitochondria. Using the same flow cytometry method and a cybrid cell line (containing wild-type and a genome with a deletion of 45% of the genome) Poe *et al.* found homoplasmic distributions within smaller nucleoids of less than 13 mtDNA copies [10]. Because the technique in both studies requires mitochondrial separation from a suspension of many cells, it is impossible to study the intracellular mtDNA distribution from a single cell with their reported methodology. Several reports describe the detection and analysis of organelles extracted from single cells [11], [12], [13] with the work of Wang and collaborators [13] being of particular interest because an optical tweezer was used to extract single chromosomes for offline genomic analysis.

In addition to trapping chromosomes, optical tweezers have also been used to trap individual mitochondria for several purposes including the study of mitochondrion transport along microtubules *in vivo* [14], the immobilization of single mitochondria for near infrared Raman spectroscopic studies [15], the extraction of individual mitochondria from isolated cells [12], [16], and the separation of individual mitochondria from a purified mitochondrial pellet extracted from *Physarum polycephalum* (slime mold) [17]. In this final example, the individual mitochondria were applied to a glass surface, dried, and the glass was cut and subjected to PCR amplification. This method required a minimum of nine mitochondria to produce a detectable amplicon [17]. References [13], [16], [17] imply that an optical tweezer-based extraction method may permit the use of single mitochondria separated from the same cell for further analysis over time.

In this paper we describe a method for isolating a single mitochondrion and sequencing its mtDNA. Here, we use this technique to verify that a heteroplasmy (a single nucleotide polymorphism [SNP]) exists within a single mitochondrion. This technique could be used to further study the propagation and properties of mitochondrial nucleoids and connections between the intracellular distribution of mtDNA mutations and the development of mitochondrial disorders. It could also be applied to the study of other organelles in cells from a single organism considered to have genetically identical cells to determine how cellular heterogeneity leads to changes in cell physiology and pathology.

## Results

### Isolation and Capture of a Single Mitochondrion Particle

**Figure 1:**
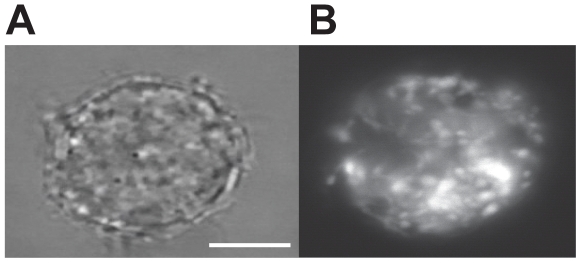
An isolated HL-60 cell. (A) A brightfield image of an HL-60 cell and (B) A confocal scan of the same cell showing mitochondria fluorescently labeled with Mitotracker Green FM. The scale bar corresponds to 5 microns.

**Figure 2:**
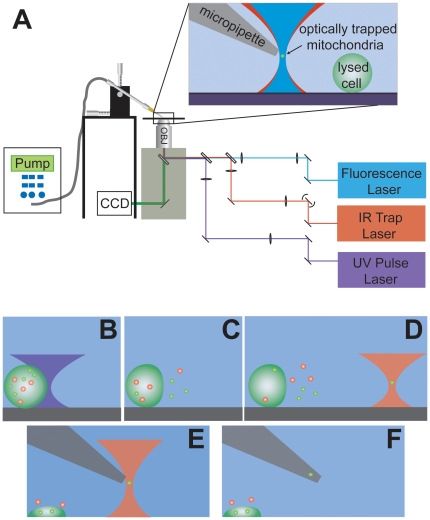
Schematic illustrations of the experimental setup and the extraction process. (A) Light from three different lasers is coupled into the back aperture of a microscope objective. The ultraviolet (UV) pulse, fluorescence, and infrared (IR) trap lasers are overlapped and used for cell lysing, mitochondrion identification and trapping respectively. A pump applies positive pressure to a femtopipette tip to balance against the capillary draw of fluid up inside the tip. (B) A single cell, isolated from other cells, is lysed with a UV pulse laser (violet hourglass). (C) Cell lysis occurs and organelles escape from the cell. (D) A trapped organelle is identified as a fluorescently-labeled mitochondrion with the fluorescence laser (IR trap is designated by the red hourglass; overlapping fluorescence excitation laser is not shown). (E) The trapped organelle is positioned near the end of a femtopipette tip. (F) The tip is positioned sufficiently close to directly capture the mitochondrion or backing pressure from the pump is removed and capillary action draws the mitochondrion into the femtopipette. Full details of the extraction protocol are described in the methods section. A time-edited video showing the entire process is found in the Supplementary Video (S1) available online.

To trap a single mitochondrion, we developed an optical tweezer based approach similar to that used for chromosome extraction from single rice cells [13]. We first isolate, by dilution, a human cell stained with a mitochondria indicator dye (Mitotracker Green FM), which specifically labels the membranes of respiring mitochondria. Figure 1 shows a brightfield and confocal fluorescence image of a typical dyed HL-60 cell. A schematic diagram of the experimental apparatus and extraction process are shown in Figure 2. The cell is lysed with one or several pulses from a Q-switched UV laser and a single-focus optical tweezer traps a freely diffusing organelle. The trapped organelle is identified as a mitochondrion with laser induced fluorescence. The trapped mitochondrion is raised above the cell to a femtopipette tip and directly inserted or suctioned, along with a small volume of buffer, into the empty tip. A time-edited video of this process is included in the supplementary material (Supplementary Video S1).

### Amplification and Sequencing mtDNA from a single mitochondrion

Extracted mtDNA from an aggregate of HL-60 cells was previously shown to contain an approximate 1∶1 C to T heteroplasmy at nucleotide position (np) 12071 [18]. We developed PCR and sequencing methodologies to examine the limited DNA contained in a single mitochondrion. Our study demonstrates that the heteroplasmy seen in the aggregate was also present in the mtDNA of the isolated mitochondrion, albeit at various ratios.

DNA amplification (PCR) was performed using two distinct primer sets (PS 1 and PS 43) from NIST Standard Reference Materials SRM 2392 and 2392-I [18], [19]. PS 43 amplifies the region containing the heteroplasmy of interest located at np 12071. This region was then sequenced to identify the presence of the heteroplasmy at the single mitochondrion level. As a further control, PS 1 amplified the hypervariable region 2 (HV2) of the mtDNA genome [20], [21]. We sequenced the HV2 region and compared this to the published sequence of HL-60 [18] to verify that the tested heteroplasmic mtDNA originated from HL-60 (Table 1).

**Table 1:** The sequence variation of the hypervariable region 2 (HV2).

NT position*	rCRS*	HL-60∧	This study∧	Sci #1	Sci #2	Sci #3	Sci #4	Sci #5
150	C	T	T	C	C	T	T	C
152	T	C	C	C	T	T	T	T
199	T	T	T	T	C	T	T	T
263	A	G	G	G	G	A	G	G
295	C	T	T	C	C	C	C	C
309	C	CC (insert)	CC (insert)	C	CC (insert)	CC (insert)	CC (insert)	CC (insert)
315	C	CC (insert)	CC (insert)	CC (insert)	CC (insert)	CC (insert)	CC (insert)	CC (insert)

*The HV2 region was sequenced between np 100–350 and compared to the revised Cambridge Reference Sequence (rCRS) [36], [37].

∧For the two samples amplified with PS 1 (single mitochondrion C and D), we found agreement with the published HL-60 sequence [18]. Sci #1 – Sci #5 refer to the five researchers that had contact with the mitochondrion or blank samples.

**Figure 3:**
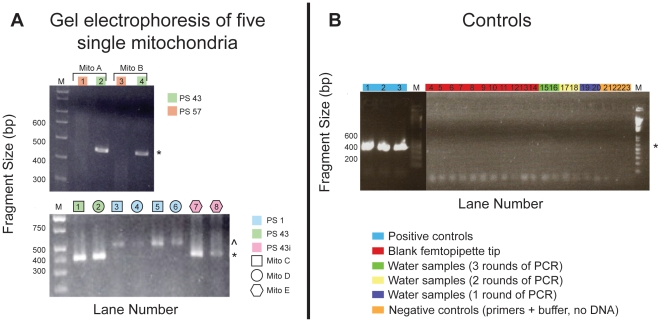
Agarose gel electrophoresis of amplified single mitochondrion samples A-E after three rounds of PCR. (A, top) Amplified product using PS 43 and single mitochondrion A and B (Lanes 2 and 4). Lanes 1 and 3 represent failed amplification with PS 57 and single mitochondrion samples A and B. (A, bottom) Amplified products with PS 43 (Lane 1) and PS 1 (Lanes 3 and 5) from single mitochondrion C. Amplified products with PS 43 (Lane 2) and PS 1 (Lanes 4 and 6) from single mitochondrion D. Amplified products from PS 43i for single mitochondrion E (Lanes 7 and 8). (B) Eleven Control A blank femtopipette tips inserted into the cell suspension for approximately 20 minutes after three rounds of PCR (Lanes 4–14) with PS 43. Water samples carried through 3 rounds (Lanes 15–16), 2 rounds (Lanes 17–18) and 1 round of PCR (Lanes 19–20.) Positive controls for each round of PCR (1.16 ng human DNA) with PS 43, one round PCR for each; Lanes 1, 2 and 3 for successive PCR rounds 1, 2 and 3 respectively. Negative controls for each round of PCR (no DNA) with PS 43, one round of PCR for each; Lanes 21–23 for round 1–3 respectively. *M* =  molecular weight size markers. Primer set 43 and PS 43i showed PCR products of approximately 0.43 kb (*), while those amplified with PS 1 showed bands of about 0.47 kb (∧).

Twenty mitochondrion particles were trapped and captured for mtDNA amplification and sequencing. A subset of the resulting PCR reactions is shown in Figure 3A. Mitochondrial DNA from five of the twenty particles (25%) was successfully amplified with PS 43 (Figure 3A). Two of these five were successfully co-amplified with PS 1 (Figure 3A), which was used to verify the identity of the mitochondrial genome as originating from HL-60 (Table 1).

To ensure the lack of PCR contamination products, a series of controls were prepared. The Control A series consisted of eleven blank femtopipette tips that were collected after being submerged for 20 minutes at a depth of 10 µm with 3 UV-lysed cells in the chamber. Control A samples were subjected to three rounds of PCR reactions. For each round of PCR reactions, one positive control (lanes 1–3, Figure 3B) and one negative control (lanes 21–23, Figure 3B) were included. In addition, two ddH_2_O controls were amplified through three rounds of PCR (lanes 15–16, 17–18, and 19–20 for rounds 1, 2 and 3 respectively, Figure 3B). Finally, the PCR amplification results of the eleven Control A femtopipette tips are shown in lanes 4–14 in Figure 3B. No amplicons were detected in any of the blank or negative ddH_2_O controls.

In a second series of controls (Control B), quantitative PCR (qPCR) was used to detect whether mtDNA was present in the media and drawn into the femtopipette tips during these experiments. Seven femtopipette tips were collected in a similar manner to the trapped mitochondrion samples and outlined in the experimental section. Control B tips were analyzed using TaqMan® AmpliTaq qPCR (Applied Biosystems, Inc., Foster City, CA) and primers that targeted the conserved cytochrome c oxidase subunit I (COI) region. The *C_t_* value refers to the qPCR cycle number (*C*) at which fluorescence is detected above a threshold value (*t*). Primer artifacts are often seen past 40 qPCR cycles, as was the case in this experiment. The average *C_t_* value for the Control B samples was above the *C_t_* values for the ddH_2_O and the no template media controls (Table 2). The qPCR amplification data can be found in the Supplementary Figure S1.

**Table 2:** Ct values for Control B samples.

Sample	C_t_ *
ddH_2_O	42.18
RPMI Media	41.77
RPMI Media	Undetected
Tip-1	47.87
Tip-2	44.10
Tip-3	46.41
Tip-4	Undetected
Tip-5	42.16
Tip-6	47.07
Tip-7	44.70

*The COI region was amplified using PS COI and TaqMan® probe COI. A series of seven dilutions were run in replicate to calculate the standard curve. Standard curve *y* = −3.628*x*−1.677, *R* ^2^ = 0.997.

**Figure 4:**
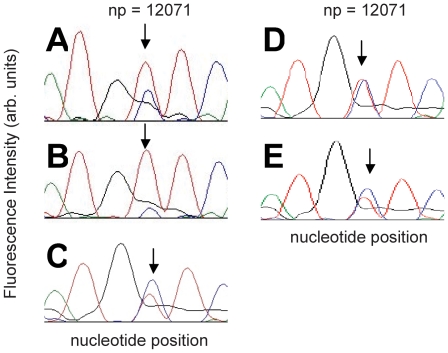
Sequencing chromatograms of single mitochondrion A–E. After optical tweezers capture, transfer and PCR amplification, the five samples that successfully amplified with primer set 43 or 43i were sequenced. Each sample showed the heteroplasmy (C nucleotide (blue) and T nucleotide (red)) at np 12071 highlighted with the arrow.

The sequencing chromatograms, resulting from the five single mitochondrion samples successfully amplified with PS 43, are shown in Figure 4. Each chromatogram is centered around np 12071. All five samples show a heteroplasmy at np 12071, albeit at different ratios. Two samples (A and B, Figure 4) contained an abundance of T, two samples (C and E, Figure 4) contained an abundance of C, while one sample (D, Figure 4) contained approximately equal ratios of C and T. While the absolute quantity is not calculated, reproducibility between PCR rounds of the same sample is demonstrated in Supplementary Figure S2.

## Discussion

Of the twenty trapped and isolated mitochondrion particles, five samples successfully amplified the SNP region of interest, and all five samples had some degree of heteroplasmy in the np 12071 position. We were able to rule out the possibility that any of the five reported samples resulted from false positives. A false positive in this case would be either the amplification of DNA from more than one mitochondria or from the wrong DNA (e.g., DNA from non-HL-60 sources or nuclear DNA). We address these concerns by noting that the primers used in these studies are mitochondrial-specific and the heteroplasmy, or simply the presence of a C homoplasmy at np 12071, is unique to the HL-60 cell line [18]. Examination of the 5,857 existing NCBI [22] whole-mtDNA sequences, MITOMAP [23] coding region variations and the mtDNA tree [24] revealed no heteroplasmy or cytosine homoplasmy at np 12071. The heteroplasmy existing at 12071 was only identified in the HL-60 cells [18]. Furthermore, the C nucleotide at 12071 was not found in any of the researchers' mtDNA (data not shown). This finding strongly implies that the three isolated mtDNA samples that amplified with PS 43 but failed with PS 1 still originated from an HL-60 cell. Our results indicate that the heteroplasmy is present at the single mitochondrial level and originated from the lysed HL-60 cell.

To establish the unlikelihood of contamination from PCR products, we amplified at least two blank control samples (ddH_2_O only) through three rounds of PCR and purification as described in the methods section for each set of femtopipette tip samples. No amplicons were detected from these blank control samples.

We also rule out the possibility of whole cell contamination because we see no evidence of amplification at the early stages of PCR analysis. Earlier work on amplifying the mtDNA from individual HL-60 cells typically showed PCR products after one round of amplification (Kishore, Levin, Deckman, and Helmerson, unpublished observations). To minimize the possibility of mitochondria or mitochondrial DNA adhering to the outside of the femtopipette tip, we kept the end of the femtopipette tip approximately 100 microns above the settled cells (cell diameter ≈10 micron) during all experiments. Also sample holders containing the cells were discarded after only one to three cell lysings. Finally, the exterior surface of the femtopipette tip is hydrophobic (Eppendorf, Inc., personal communication, Westbury, NY). Thus, the polar membrane surfaces of cells and mitochondria or its DNA are unlikely to adhere to the outside of the femtopipette tip.

In addition to these experimental design controls, we characterized the likelihood of mitochondria adhering to the outside of the femtopipette tip by performing “blank-tip” controls. Eleven femtopipette tips (described above as series Control A) were submerged in solutions containing HL-60 cells at experimentally relevant cellular concentrations. Three cells were then lysed with UV laser pulses and the femtopipette tip was maintained 100 microns above the coverglass surface for a period of 20 minutes within the vicinity of the three lysed cells. If the five positive results occurred because of unwanted mitochondria adhering to the outside of the femtopipette tip, then the 25% success rate (assumes each mitochondria contains at least one mtDNA copy) implies that we should see two to three blank-tip positives (binomial distribution). We observed no amplicon production, which had a 4.2% chance of occurring given a 25% success rate from any of the eleven blank tips. This suggests, with greater than 95% confidence that our five positive samples did not result from excess mitochondria non-specifically binding to the outside of the femtopipette tip.

To minimize the possibility that a free mitochondrial DNA molecule diffused through the media to the femtopipette tip and was drawn into the tip alone or with a mitochondrial particle, the cell was lysed and a mitochondrion was collected over a maximum time frame of twenty minutes. Second, quantitative real-time PCR studies were conducted as follows: seven femtopipette tips (as described above as Control B experiments) were collected following the standard protocol with one modification. A single, optically trapped mitochondrion particle was raised to the femtopipette tip and then released by blocking the trapping beam. The mitochondrion particle was observed to float away. After which, a parcel of media blank solution (≈ pL's) was drawn into the tip. The average *C_t_* value for each sample was greater than or equal to our no template control (ddH_2_O and media) samples (see Supplementary Figure S2).

The inability of the five successful samples to amplify with both PS 43 and PS 1 was most likely due to the very limited amount of mtDNA and stochastic effects with the amplification reaction. Cavelier *et al.* reported that the average mtDNA genome copy number in a single mitochondrion was around two and ranged from zero to eleven copies [9]. In addition, mtDNA was not detected in 41 to 53% of collected mitochondria (Cavelier *et al.,* [9]). A more recent flow cytometry study, with a reported PCR efficiency of 80%, found that 35% of the mitochondria, containing mtDNA, showed no amplified PCR product [10]. Both studies suggest various technical issues that minimize the success of flow cytometry studies. Therefore, our trapping and PCR amplification success rate, albeit lower than these studies, is not unreasonable.

Our results are reasonably consistent with the reported work of Cavelier et al. [9] where approximately 50% of the isolated mitochondria contained no detectable mtDNA, approximately 13% contained heteroplasmic mixtures of wild-type and MERRF (SNP) genomes, and the remaining 37% contained homoplasmic distributions. Although we see no homoplasmic mitochondria in our five samples that amplified, they note that approximately 18% of the cells from their study were homoplasmic to begin with. This, along with the fact that mitochondria containing a low copy number of mtDNA genomes tend to bias towards homoplasmic distributions [9], partially explains the higher rate of homoplasmic detection they report.

Mitochondria *in vivo* have various morphologies ranging from spheres (in HL-60 cells and neutrophils [25]) to branched tubular networks (e.g. [26]; for reviews see references [27] and [28]). The fibroblast cell line employed by Cavelier *et al.* are elongated and networked [9]. They emphasized, though, that through mitochondrial fission, their manipulation of the cells and their sorting selection process prevented elongated or larger mitochondrial particles from being selected. We expect that our method will self select a spherical particle as well. Nevertheless, even though many organelles are not spherical in shape, this should not hinder the technique described herein because single focus optical tweezers have been shown to trap non-spherical objects including semiconducting nanowires [29], [30], rod-shaped cilium [31] and chromosomes [13], [32], [33]. Line scan optical tweezers [34] and holographic optical trapping [35] are two examples of modifications that have also been used to trap non-spherical particles.

In this paper, we employed an optical tweezer based method [13] for extracting single mitochondria from UV lysed cells with an optical tweezer and femtopipette tip. Optical tweezers separate the mitochondria from the vicinity of the lysed cell and a single focus fluorescence laser identifies the organelle, which had been stained with Mitotracker Green FM, as a mitochondrion particle. The single mitochondrion is transferred from the femtopipette tip to a centrifuge tube followed by lysing, PCR amplification, and sequencing. This work indicates that a heteroplasmy in the mtDNA, previously shown in our laboratory to exist at the single cell level, also exists at the single mitochondrion level. By applying this technique to multiple mitochondria from the same cell, it should be possible to measure the intracellular distribution of a mtDNA mutation and learn what role, if any, this distribution plays in the development of mitochondrial-based diseases. Finally, the optical tweezer method described herein along with qPCR fluorescence analysis could be used to accurately quantify genome copy numbers and mutant to wild-type ratios of mtDNA.

## Methods

### Chemicals

Mitotracker Green FM and oligonucleotide primers as described in Table 3 were purchased from Invitrogen, Inc. (Carlsbad, CA). *Taq* DNA polymerase was purchased from Promega, Inc. (Madison, WI). All other chemicals were purchased from Sigma-Aldrich, Inc. (St. Louis, MO) unless otherwise noted.

**Table 3:** Primer sequences used for PCR, sequencing and qPCR.

Primer Set (PS)	Nucleotide Position*	Sequence∧
1F	15	5′ CACCCTATTAACCACTCACG 3′
1R	484	5′ TGAGATTAGTAGTATGGGAG 3′
43F	11760	5′ ACGAACGCACTCACAGTCG 3′
43R	12189	5′ AAGCCTCTGTTGTCAGATTCAC 3′
43iF	11779	5′ CATCATAATCCTCTCTCAAGG 3′
43iR	12176	5′ AATCTGATGTTTTGGTTAAAC 3′
57F	15971	5′ TTAACTCCACCATTAGCACC 3′
57R	16451	5′ GCGAGGAGAGTAGCACTCTTG 3′
COIF	6048	5′ GGTAACGACCACATCTACAACGTT 3′
COIR	6134	5′ GCCTCCGATTATGATGGGTATTACT 3′
COI-Probe	6074	FAM-CGTCACAGCCCATGCATTTGTAATAATCTTCTTC-TAMRA

*The nucleotide position in the mtDNA genome is based on the rCRS [36], [37].

∧ Primer sets 1, 43 and 57 are based on the SRM-2392-I by Levin *et al*. [18].

### Cell line

The promyelocytic leukemic cell line HL-60 consisting of peripheral blood leukocytes from a 36-year-old woman (ATCC CCL-240) was maintained in RPMI growth medium without glutamine (containing 10% fetal bovine serum, 100 µg/mL penicillin G, 100 µg/mL streptomycin sulfate and 0.25 µg/mL amphotericin B) at 37°C, 5% CO_2_ and humidity. One milliliter of this culture was pelleted at 14,000 RPM centrifugation for 1 min, and the pellet was resuspended in 1 mL of growth medium (RPMI-1640 described above) containing 0.1 µmol/L Mitotracker Green FM in DMSO. The cells were incubated (37°C, 15 min), pelleted again, and washed (2x in 1 mL fresh growth media). Cells were resuspended in 1 mL of fresh growth media at an appropriate dilution, identified below, and stored at 4°C in preparation for the optical tweezer extraction.

### Isolation and Capture of a Single Mitochondrion Particle

A 120 microliter well was created by mounting a #0 coverslip with vacuum silicone grease to a microscope slide containing a 1 cm diameter hole. Ten microliters of the previously prepared HL-60 culture were mixed with 110 µL of isotonic PBS buffer (137 mmol/L NaCl, 10 mmol/L potassium phosphate, 2.7 mmol/L KCl, pH 7.2), and the solution was transferred to the well. The cells in solution were allowed to settle onto the glass cover slip for 10 minutes. The cells were observed to remain intact and did not swell or lyse for the duration of the experiment. The settled cell concentration was on the order of 50 cells/mm^2^. Three overlapped, collimated, laser beams were reflected off a dichroic mirror (505DRLP, XF2010, Omega Optical, Brattleboro, VT) and sent through the back aperture of an oil-immersion 100× microscope objective (NA  = 1.3, Plan Neofluar, Carl Zeiss, Thornwood, NY) mounted on an inverted microscope (Axiovert 100, Carl Zeiss). Individual cells were lysed with a pulsed (pulse duration ≈5 ns, pulse energy ≈50 µJ) UV laser operating in triggered single pulse mode at 355 nm (ML-II, Continuum, Inc., Santa Clara, CA). A continuous wave (CW) infrared (IR) laser operating at 500 mW and 1064 nm (YLD-10-LP, IPG Photonics, Oxford, MA) was used to optically trap each mitochondrion particle. Each trapped particle was identified as a Mitotracker Green FM labeled mitochondrion by fluorescence excitation from an Ar^+^ laser (CVI Melles Griot, Albuquerque, NM) operating at 488 nm and approximately 5 mW–10 mW. Emitted fluorescence from the trapped mitochondria was collected back through the 100× objective and focused onto a CCD camera (Sunstar 300, Electrophysics Inc., Fairfield, NJ) mounted onto the microscope. The presence of more than one organelle in the trap could be seen at the video rate (15 Hz) under bright field illumination. If more than one mitochondria was visualized or the trapped organelle did not fluoresce with 488 nm excitation, then the IR tweezer laser was blocked and the contents of the trap were allowed to diffuse away. A recent study reported the use of a 1064 nm optical tweezer leads to two-photon fluorescence and subsequent photobleaching of MitoTracker Green labeled mitochondria over a period of 5 seconds [16]. We do not observe this rapid degree of photobleaching in our system. Photobleaching of this magnitude would lead to a prohibitive number of false negatives (rejected mitochondria) in our protocol. The supplemental video shows a time-edited sequence of the trapping and fluorescence based identification steps and very little detectable 1064 nm induced photobleaching. The protocol was repeated until a single mitochondrion was trapped.

To acquire a mitochondrion particle for genotyping analysis, a hydrophobically treated femtopipette tip (Eppendorf, Inc. personal communication, Westbury, NY) with an attached pump (Femtojet, Eppendorf Inc., Westbury, NY) applying a continuous positive pressure of approximately 1 atm to prevent capillary suction of fluid, was positioned less than 1 micron from the trapped mitochondrion with a micromanipulator (MP-285, Sutter Instrument Co., Novato, CA). The positive pressure was reduced over a period of 2-3 seconds while the IR tweezer laser was blocked; this resulted in the mitochondrion being drawn into the tip. In a few cases, the suction step was unnecessary because the femtopipette tip was positioned sufficiently close to the trapped mitochondrion and the mitochondrion was directly captured by the end of the femtopipette tip (see Supplementary Video S1). The rate of success of capture was dependent upon the distance and position of the mitochondrion from the center of the tip. Approximately 75% of the trapped single mitochondria were drawn into the tip. To the best of our knowledge, each tip contained one trapped mitochondrion. The contents of the femtopipette tip were transferred into a sterile 250 µL centrifuge tube containing 10 µL sterile deionized distilled H_2_O (ddH_2_O, Gibco-BRL, Gaithersburg, MD) by touching the end of the tip to the bottom of the tube. A fragment of the glass femtopipette tip was intentionally broken which resulted in a capillary draw of buffer into the broken tip. Several positive pressure pulses (1 atm applied for 100 ms) flushed this excess solution back into the tube. The single mitochondrion sample was then stored in a −20°C freezer until further analysis could be performed. This process was repeated with approximately 1-3 well-separated cells before discarding the slide and repeating with a fresh sample of cells.

To demonstrate that the hydrophobic exterior surface of the femtopipette tip did not bind free mtDNA or mitochondrion particles, Control A experiments were completed. Eleven femtopipette tips were submerged in solutions containing HL-60 cells at experimentally relevant cellular concentrations. Three cells were then lysed with UV laser pulses and the femtopipette tip remained 100 microns above the coverglass surface for a period of 20 minutes within the vicinity of the three lysed cells. The contents of these eleven blank negative control tips were transferred to a sterile centrifuge tube in a manner similar to that highlighted above.

To determine whether free (intact or fragmented) mtDNA or unidentified mitochondrion particles were drawn into the tip with the trapped mitochondrion, seven buffer-only tip controls (Control B) were collected utilizing the same protocol as described in the capture section except that the IR trapping beam was blocked after the mitochondria had been identified and positioned near the end of the femtopipette tip. The trapped mitochondrion particle was allowed to diffuse away and the backing-pressure was lowered to allow only the buffer to be drawn up into the tip.

### PCR Protocol

Each sample consisting of the end piece of the femtopipette tip and its single mitochondrion (or tips from Controls A or B) was sonicated (Sonifier 450, Branson, Danbury, CT) with 120 W, 2-second pulses for 2 minutes. Heat (95°C, 10 min) was then used to continue to disrupt the mitochondrion membranes. The single mitochondrion sample posed two general problems. First, a single mitochondrion sample could not be divided in order to complete the independent amplification of two regions. Second, the mtDNA genome copy number in a mitochondrion is unknown at the time of sampling and can vary from zero to 10 or more [9]. Therefore, in order to successfully amplify these samples mitochondrial DNA was subjected to three rounds of PCR using standard conditions and 2.5 units *Taq* DNA polymerase (Promega Corp., Madison, WI) following the manufacturer's protocol. Typical PCR parameters were: 95°C for 10 min, 35 cycles (94°C for 20 sec, 50°C for 20 sec, 72°C for 40 sec), and 72°C for 7 min. Each primer concentration was 200 nmol/L. All PCR reactions were analyzed with 2% agarose gels in TBE (100 mmol/L Tris, 90 mmol/L boric acid and 1 mmol/L EDTA, pH 8.3) and stained with 0.5 µg/mL ethidium bromide. A thermocycler (9700 or 2400, Perkin Elmer Inc., Waltham, MA) was used for all PCR amplifications. Primer sequences are highlighted in Table 3, which includes the nucleotide position for the 5′ end of the primer within the mtDNA genome [36], [37].

Initially, primer set (PS) 43 and either PS 1 or PS 57 were added to the single mitochondrion sample. After each round of PCR, the amplicons were purified using QIAquick PCR columns (Qiagen, Inc., Valencia, CA) according to the manufacturer's protocol. The PCR products were eluted with 50 µL ddH_2_O. Aliquots (1 to 10 µL) of the first and second rounds of purified amplicons were used in subsequent PCR reactions to determine the best method for PCR amplification success. Primer combinations and volume used in the series of PCR reactions are available in Table 4 and are summarized here. In Round 1, both sets of primers (PS 43 and PS 57 or PS 1) were co-amplified. Five or ten µL of Round 1 amplicon (not visible by agarose gel electrophoresis) were reamplified in Round 2. One, five or ten µL of the Round 2 PCR product (not visible by agarose gel electrophoresis) were reamplified in Round 3. Samples A and B had co-amplification of PS 43 and PS 57 in Round 2, while samples C, D and E were divided and PS 43 and PS 1 were amplified independently. Sample E utilized PS 43i, a nested primer set, for Rounds 2 and 3. PS 57 was used in earlier captured mitochondria but it failed to amplify any sample. Subsequent samples were amplified with PS 1.

**Table 4:** Sample manipulation for PCR amplification of mtDNA from a single mitochondrion.

Sample	PCR Round	Template	Final Reaction Volume	Primers
A	1	Single mitochondrion A in 30 µL dH_2_O	50 µL	PS43 and PS57
	2	10 µL round 1 amplicon	50 µL	PS43 and PS57
	3	10 µL round 2 amplicon	50 µL	PS43 or PS57
B	1	Single mitochondrion B in 30 µl dH_2_O	50 µL	PS43 and PS57
	2	10 µL round 1 amplicon	50 µL	PS43 and PS57
	3	10 µL round 2 amplicon	50 µL	PS43 or PS57
C	1	Single mitochondrion C in 10 µl dH_2_O	25 µL	PS43 and PS1
	2	5 µL round 1 amplicon	25 µL	PS43 or PS1
	3	1 µL round 2 amplicon	25 µL	PS43 or PS1
D	1	Single mitochondrion D in 10 µl dH_2_O	25 µL	PS43 and PS1
	2	5 µL round 1 amplicon	25 µL	PS43 or PS1
	3	5 µL round 2 amplicon	25 µL	PS43 or PS1
E	1	Single mitochondrion E in 10 µl dH_2_O	25 µL	PS43 and PS1
	2	5 µL round 1 amplicon	25 µL	PS43i or PS1
	3	5 µL round 2 amplicon	25 µL	PS43i or PS1

The eleven blank Control A samples and ddH_2_O blanks (no tip) were analyzed in an identical manner, including three rounds of amplification and purification of each PCR reaction between amplifications. At a minimum, two blank ddH_2_O samples were analyzed for each series of femtopipette tip experiments.

The seven buffer blank tip samples from Control B were subjected to quanititative PCR (qPCR) analysis utilizing a TaqMan® (Applied Biosystems, Inc.) probe and primers designed to amplify a region within the highly conserved cytochrome c oxidase subunit I gene (COI). FastStart TaqMan® Probe Master Mix (Roche, Inc., Indianapolis, IN) was used following the manufacturer's procedure. The primers (400 nmol/L each) and probe (200 nmol/L) sequences are identified in Table 3. The probe was labeled with fluorescamine (5′-FAM) and the quencher 3′-TAMRA. Typical qPCR conditions were: 50°C for 2 min, 95°C for 10 minutes, and 50 cycles (95°C for 15 sec and 60°C for 1 min.)

All positive PCR amplification controls contained the appropriate primer set and 1.6 ng HL-60 total DNA (CCL 240D, ATCC, Manassas, VA). All PCR negative controls contained the appropriate primer set but lacked any amplifiable DNA. These positive and negative PCR controls reactions were prepared in separate laboratory spaces isolated from the extracted tip samples and tip controls to prevent contamination. DNA samples taken from researchers, involved in the trapping or amplification procedure, were amplified and sequenced with PS 1 and PS 43. All DNA sequences were compared to the revised Cambridge Reference Sequence (rCRS) [37], [38].

Any negative water controls that contained evidence of contamination were discarded and a new set of samples was collected. These discarded samples are not included in any calculation demonstrating overall success.

### DNA sequencing protocol

One to five microliters of each PCR reaction was mixed with 0.5 µmol/L of the appropriate forward or reverse primer and 8 µL BigDye Terminator v1.1 Cycle Sequencing Kit according to the manufacturer's protocol (Applied Biosystems, Inc., Foster City, CA). Sequencing reactions were utilized the following thermocycler parameters: 95°C for 2 min and 25 cycles (95°C for 15 sec, 50°C for 5 sec, and 60°C for 2 min). Products were purified using the Performa DTR gel cartridge (Edge Biosystems, Inc., Gaithersburg, MD) and dried in a vacuum microcentrifuge (SpeedVac, Thermo Fisher Scientific, Waltham, MA). Twenty µL of Template Suppression Reagent (Applied Biosystems, Inc.) or formamide was added to each sample and analyzed by capillary gel electrophoresis (Genetic Analyzer 310, Applied Biosystems, Inc.). Sequence Navigator v1.0.1 (Applied Biosystems, Inc.) was used for sequence alignment.

The successful sequencing of mtDNA from the five samples shows that the UV laser had no detectable damaging effect on the mtDNA.

## Supporting Information

Supplementary Figure S1 The log of fluorescence intensity, which is proportional to the concentration of amplified DNA, is plotted against the number of PCR amplification cycle (C). The fluorescence threshold (t) for detecting mtDNA is set to 0.2 (orange solid line) as determined with a standard curve. The cycle at which fluorescence reaches the threshold value is Ct. Blank controls consisted of distilled water (purple line) and RPMI media (light blue line). Each shows a Ct above 41 cycles. Two (red and orange lines) of the seven blank tips for control B (see main text) and four other blank Control B tips (not shown) show similar results with Ct values between 42 and 48 cycles. One blank tip and one media control sample did not have detectible products (see Table 2 in main text). This suggests the buffer drawn into the femtopipette tip for the Control B samples "amplify" below the limit of detection of the qPCR analysis.
(10.75 MB TIF)

Supplementary Figure S2 Chromatograms A–D display the sequencing results of a single mitochondria (mitochondrion A from figure 4 of the main text) measured after subsequent rounds of PCR. The black arrows indicate the heteroplasmic nucleotide position 12071. Chromatograms E–F show subsequent rounds of PCR on a single cell, which also shows no discernable change of heteroplasmic ratio from multiple rounds of PCR. Note the vertical axis is the same between A–D and only a slight shift in the vertical axis exists between E and F, which has little affect on the heteroplasmic ratio.
(5.29 MB TIF)

Supplementary Video S1 Time-edited video of the mitochondrion extraction process. A UV laser pulse lyses a single HL-60 cell open. An organelle diffuses away from the lysed cell. The microscope stage is adjusted so the IR optical tweezer laser, fixed in the middle of the screen, traps the organelle. The bright field light is blocked and the 488 nm fluorescence laser, overlapped with the tweezer laser, illuminates the organelle to verify it is a mitochondrion particle. The bright field light is turned back on and the femtopipette tip is positioned to capture the mitochondrion. After capture the fluorescence laser is turned back on and the femtopipette tip is moved in a controlled fashion to verify the mitochondrion is trapped inside the femtopipette tip.
(21.23 MB MOV)
