# Supplementary figures and images for: Detection of Heteroplasmic Mitochondrial DNA in Single Mitochondria
Source: PLoS One. 2010 Dec 16;5(12):e14359. doi: 10.1371/journal.pone.0014359 (PMC3002942; doi:10.1371/journal.pone.0014359)

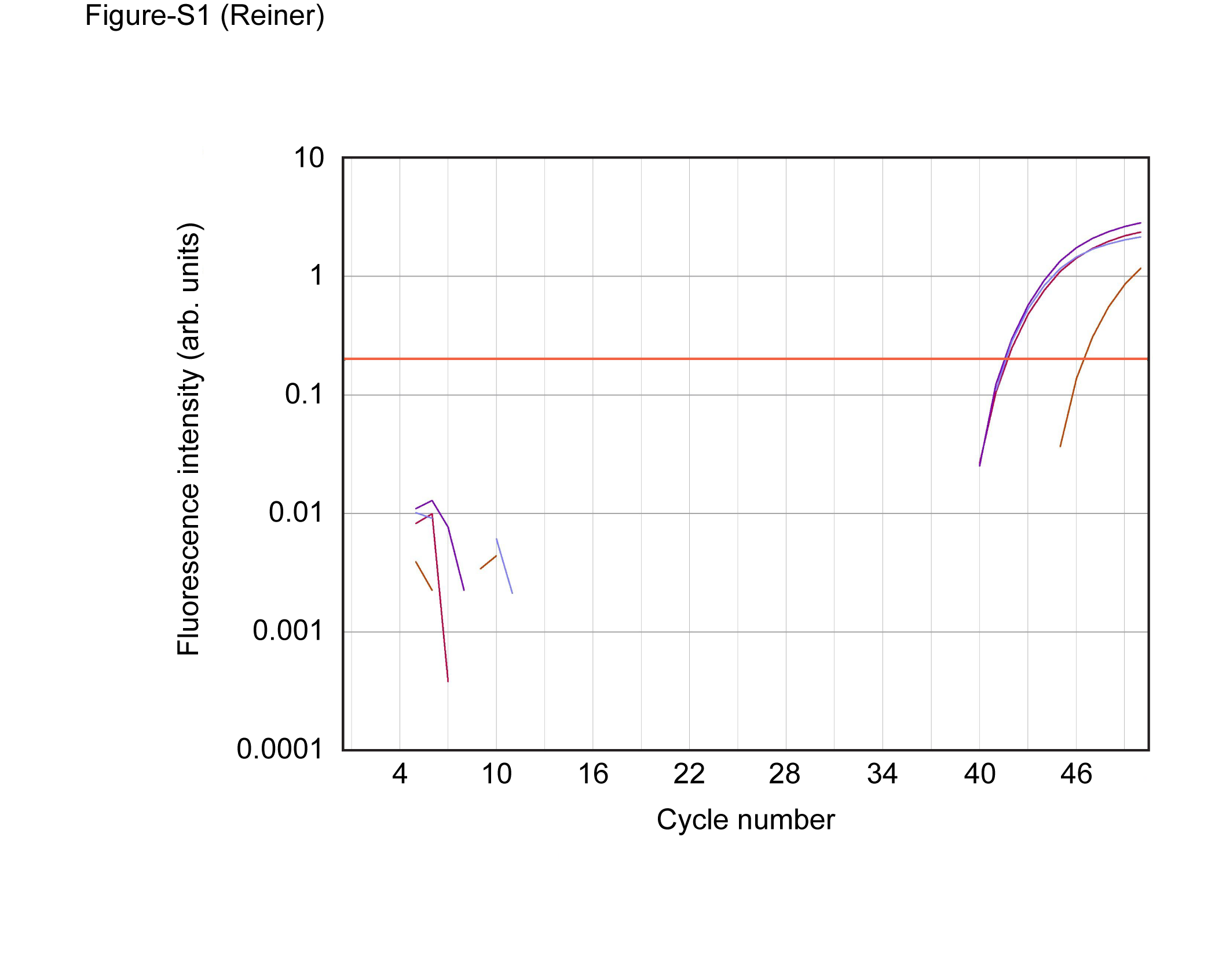

Supplement: Supplementary Figure S1 — The log of fluorescence intensity, which is proportional to the concentration of amplified DNA, is plotted against the number of PCR amplification cycle (C). The fluorescence threshold (t) for detecting mtDNA is set to 0.2 (orange solid line) as determined with a standard curve. The cycle at which fluorescence reaches the threshold value is Ct. Blank controls consisted of distilled water (purple line) and RPMI media (light blue line). Each shows a Ct above 41 cycles. Two (red and orange lines) of the seven blank tips for control B (see main text) and four other blank Control B tips (not shown) show similar results with Ct values between 42 and 48 cycles. One blank tip and one media control sample did not have detectible products (see Table 2 in main text). This suggests the buffer drawn into the femtopipette tip for the Control B samples "amplify" below the limit of detection of the qPCR analysis. (10.75 MB TIF) [file pone.0014359.s001.tif]

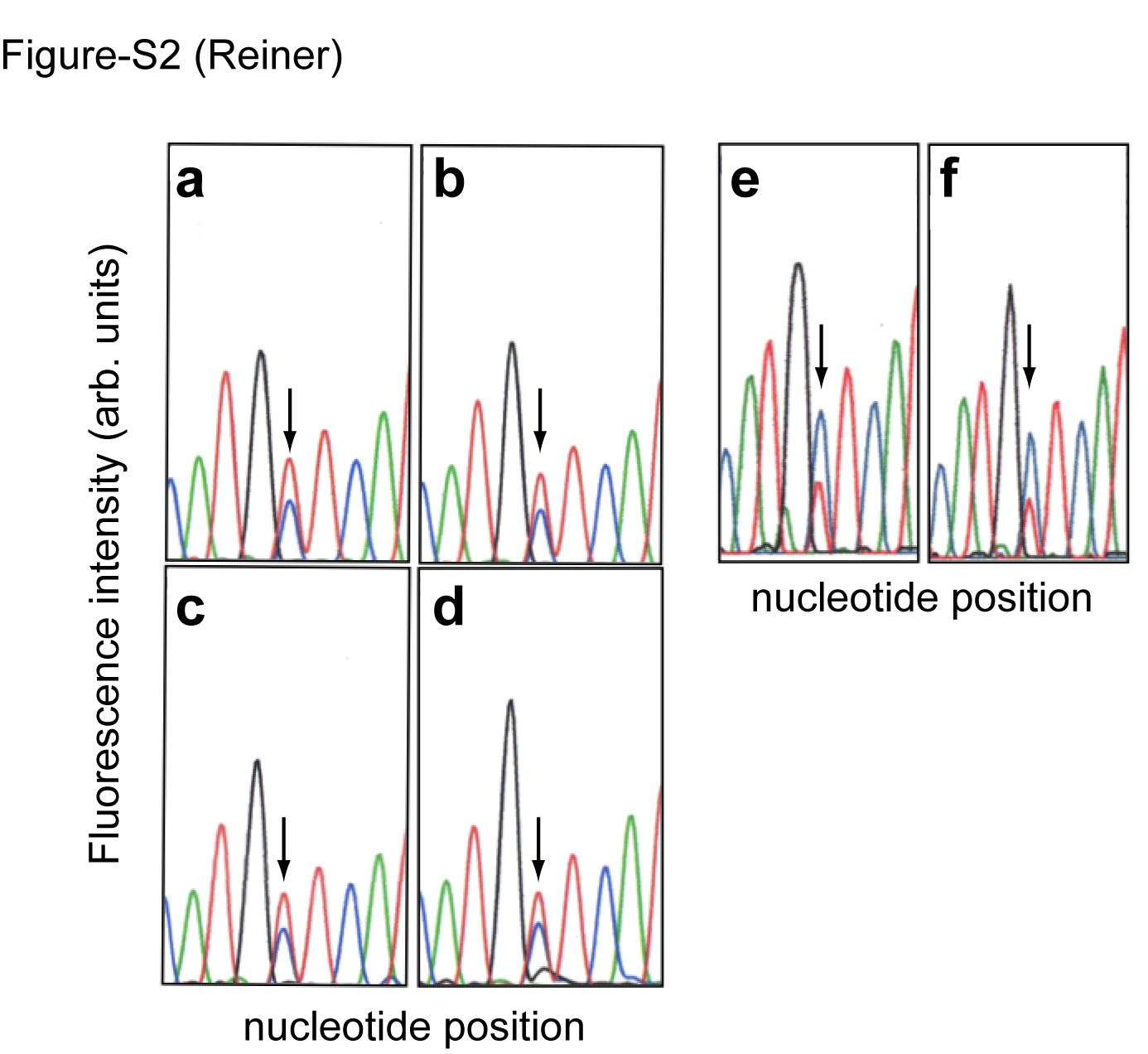

Supplement: Supplementary Figure S2 — Chromatograms A–D display the sequencing results of a single mitochondria (mitochondrion A from figure 4 of the main text) measured after subsequent rounds of PCR. The black arrows indicate the heteroplasmic nucleotide position 12071. Chromatograms E–F show subsequent rounds of PCR on a single cell, which also shows no discernable change of heteroplasmic ratio from multiple rounds of PCR. Note the vertical axis is the same between A–D and only a slight shift in the vertical axis exists between E and F, which has little affect on the heteroplasmic ratio. (5.29 MB TIF) [file pone.0014359.s002.tif]
